# Editorial: Post-transcriptional modifications in cancer immunity and immunotherapy

**DOI:** 10.3389/fimmu.2025.1680859

**Published:** 2025-08-21

**Authors:** Gaigai Wei, Yufeng Zhou, Tao Yue, Duanwu Zhang

**Affiliations:** ^1^ Children’s Hospital of Fudan University, National Children’s Medical Center, and Shanghai Key Laboratory of Medical Epigenetics, International Co-laboratory of Medical Epigenetics and Metabolism, Ministry of Science and Technology, Institutes of Biomedical Sciences, Fudan University, Shanghai, China; ^2^ National Health Commission (NHC) Key Laboratory of Neonatal Diseases, Fudan University, Shanghai, China; ^3^ Center for the Genetics of Host Defense, The University of Texas Southwestern Medical Center, Dallas, TX, United States

**Keywords:** post-transcriptional modification, alternative splicing, splicing factor, RNA methylation, neoantigen, tumor microenvironment, cancer immunity, immunotherapy

Post-transcriptional modifications (PTMs) of RNA represent an essential and highly dynamic layer of gene regulation, encompassing mechanisms such as alternative splicing, RNA methylation (e.g., m^6^A, m^5^C, m^7^G), editing, and chemical modifications like poly-ADP-ribosylation (PARylation) (1–3). These modifications do not merely diversify the transcriptome; they also reprogram cellular identity and function, especially in the context of cancer (4–6). In recent years, mounting evidence has revealed that PTMs profoundly influence tumor immunity—governing immune recognition, antigen processing, and microenvironment remodeling (7, 8). Accordingly, this Research Topic brings together six contributions, including three original research articles, two mini reviews, and one comprehensive review, to advance our understanding of the interplay between PTMs and cancer immunity and to highlight emerging opportunities for immunotherapy.

## Splicing regulators as modulators of tumor–immune dynamics

Two original research articles in this Topic delve into the role of RNA splicing regulators in orchestrating immune evasion and tumor progression. In their study, Ren et al. identify GPATCH3 as a previously uncharacterized G-patch domain protein that enhances the ATPase activity of the spliceosomal helicase DHX15, thereby modulating alternative splicing fidelity. Elevated GPATCH3 expression was associated with poor prognosis across cancer types and an immunosuppressive tumor microenvironment (TME), including increased infiltration of MDSCs and cancer-associated fibroblasts and reduced cytotoxic T and NK cells. Mechanistically, GPATCH3 deficiency led to altered splicing and expression of immune-related genes such as *CXCR3*, *CD44*, and *FOXP3*, suggesting that dysregulated splicing underlies immune escape. These findings uncover a novel axis—GPATCH3–DHX15—that functionally connects splicing machinery to immunoregulation and represents a potential therapeutic target.

Similarly, Zhao et al. focus on TSSC4, a spliceosome-associated protein with tumor-suppressive functions. Their data show that TSSC4 deficiency disrupts alternative splicing programs involved in cell cycle regulation and apoptosis, promoting tumor cell proliferation and migration. Although this study does not directly investigate immune consequences, it reinforces the principle that splicing fidelity is tightly linked to oncogenic reprogramming and may indirectly influence immune recognition.

## Multi-omics analysis reveals splicing-driven tumor subtypes

In a complementary study, Liu et al. employ an integrated transcriptomic and single-cell approach to dissect the alternative splicing landscape in diffuse midline glioma (DMG)—a pediatric brain tumor characterized by the H3K27M mutation. Their findings highlight widespread splicing alterations associated with neural regulation, immune signaling, and nucleotide metabolism. Notably, the authors identify a subset of splicing-regulated genes (*OBSCN*, *RGL1*, *ARHGEF9*, among others) that define transcriptionally and prognostically distinct tumor subtypes—one enriched for neural features and another for immune-related gene expression. Among these, *RALYL* emerged as a potential driver of stemness and immune modulation. This study not only expands the known repertoire of PTM-related changes in DMG but also proposes a splicing-based stratification framework with therapeutic relevance.

## RNA chemical modifications and immune regulation

Beyond splice site selection, PTMs involving chemical modifications of RNA also play crucial roles in cancer immunity. Zhang et al. provide a comprehensive review of the m^6^A methyltransferase METTL3 and its multifaceted roles in immune regulation. They discuss how METTL3-mediated methylation modulates dendritic cell activation, macrophage polarization, and T cell exhaustion—highlighting its emerging value as a biomarker and immunotherapeutic target. The review also synthesizes findings from various tumor types, offering a broad perspective on how m^6^A methylation can either promote or suppress anti-tumor immunity depending on context.

In a related vein, Matsumoto et al. present a mini review on PARylation, a reversible modification not only of proteins but also of RNA. The authors explore how PARylation impacts innate immune signaling pathways, including DNA damage responses and cGAS-STING activation, thereby influencing tumor immune resistance. This article draws attention to a less commonly studied form of RNA-related PTM and argues for its inclusion in future immunotherapy strategies.

## Splice variant–derived neoantigens: a frontier for immunotherapy

Huang et al. contribute a mini review that bridges the mechanistic and translational aspects of splicing. They examine how tumor-specific alternative splicing events generate neoantigens, which can be presented by MHC molecules and recognized by T cells. The review highlights computational pipelines for neoantigen prediction, recent advances in splicing-derived epitope identification, and potential applications in personalized cancer vaccines and adoptive T cell therapy. This work underscores the untapped potential of splicing-derived immunopeptidomes in precision oncology.

## Conclusion and future directions

**Figure 1:**
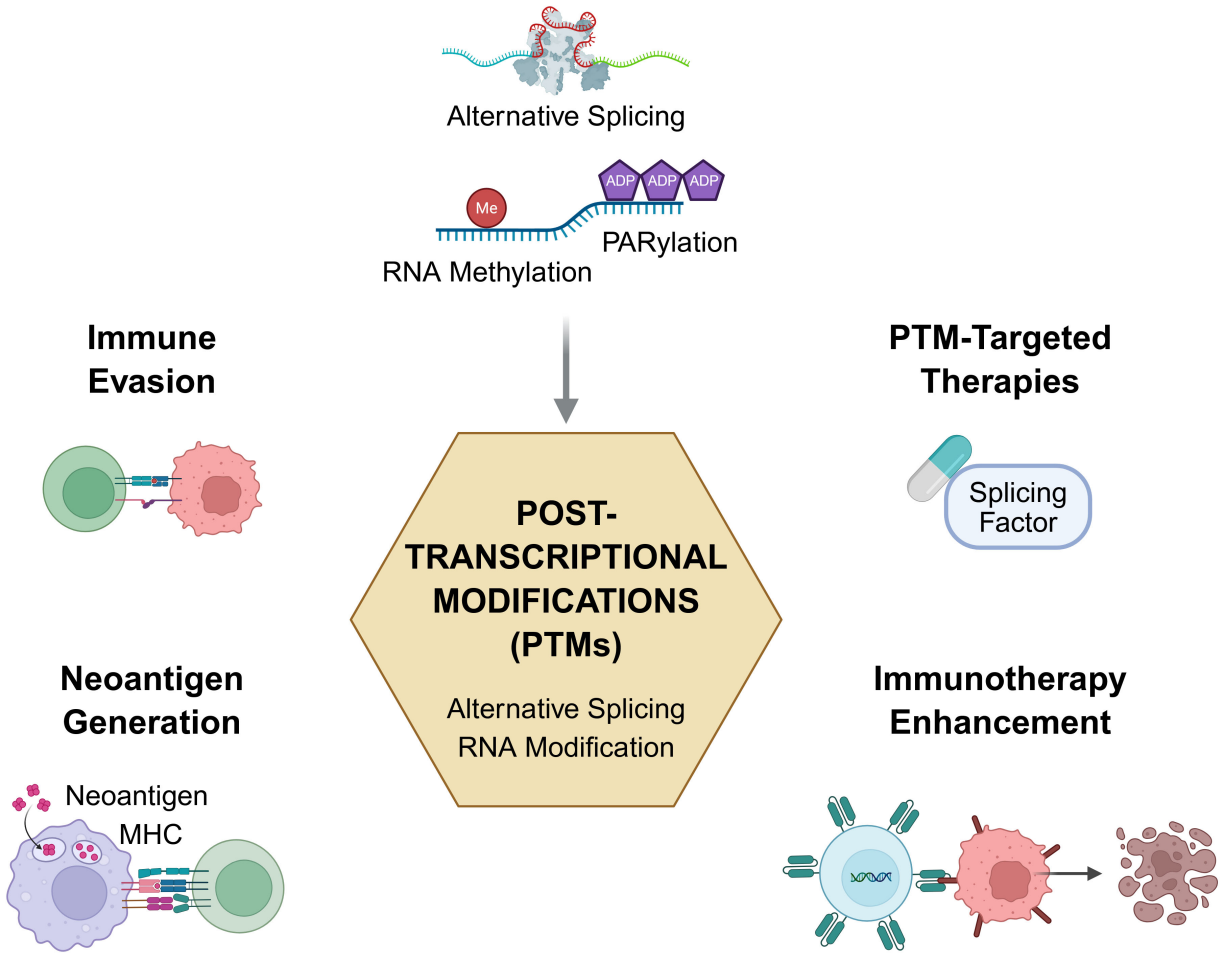
Post-transcriptional modifications in cancer immunity and immunotherapy. Schematic illustrating the critical role of post-transcriptional modifications (PTMs)—including alternative splicing, RNA methylation, RNA editing, and poly-ADP-ribosylation (PARylation)—in shaping tumor immunity and informing therapeutic strategies. PTMs orchestrate four key immunological and oncogenic processes (1): Immune evasion, by altering the splicing or expression of immune-regulatory genes; (2) Neoantigen generation, through tumor-specific splice variants that yield novel MHC-presentable epitopes; (3) PTM-targeted therapies, via modulation of splicing factors or RNA-modifying enzymes; and (4) Immunotherapy enhancement, by reshaping the tumor microenvironment or synergizing with immune checkpoint blockade. Articles featured in this Research Topic highlight these functional links between RNA regulation and cancer immunity, offering new directions for precision immunotherapy. Illustration was created using BioRender.

Together, the contributions in this Research Topic illuminate the multifaceted roles of post-transcriptional modifications in shaping cancer immunity. From spliceosomal cofactors like GPATCH3 and TSSC4 to chemical modifiers such as METTL3 and PARylation enzymes, PTMs emerge not only as passive molecular consequences but as active drivers of immune phenotypes and therapeutic vulnerabilities (**Figure 1**).

Looking forward, several challenges and opportunities remain. First, deciphering the spatiotemporal dynamics of PTMs at single-cell and spatial resolution will be key to understanding their role in TME heterogeneity. Second, the development of high-throughput functional assays to validate PTM-mediated immune regulation is urgently needed. Lastly, integrating PTM-targeting strategies with immune checkpoint blockade or neoantigen-based therapies offers promising avenues to overcome immune resistance and personalize treatment (**Figure 1**).

We hope this Research Topic will inspire continued investigation into post-transcriptional modifications as both mechanistic drivers and actionable targets in cancer immunology.
